# Surgical Management of Complex Ankle Fractures in Patients with Diabetes: A National Retrospective Multicentre Study

**DOI:** 10.3390/jcm13133949

**Published:** 2024-07-05

**Authors:** Raju Ahluwalia, Caeser Wek, Thomas Lorchan Lewis, Thomas David Stringfellow, Duncan Coffey, Sze Ping Tan, Michael Edmonds, Marco Meloni, Ines L. H. Reichert

**Affiliations:** 1 Diabetic Foot Clinic, Kings College Hospital, London SE5 9RS, UK; michael.edmonds@nhs.net; 2 Department of Orthopaedics, Kings College Hospital, Bessemer Road, London SE5 9RS, UK; c.wek@nhs.net (C.W.); thomas.stringfellow@doctors.org.uk (T.D.S.); duncan.coffey@nhs.net (D.C.); szeping.tan@nhs.net (S.P.T.); ines.reichert@kcl.ac.uk (I.L.H.R.); 3 The HARnT Collaborative King’s College Hospital, London SE5 9RS, UK; 4 Department of Diabetic Foot Unit, University of Tor Vergata, 00133 Roma, Italy; meloni.marco@libero.it

**Keywords:** ankle fracture, diabetes, osteosynthesis, neuropathy, observational study, outcomes, hindfoot nail, complications, level of evidence: III

## Abstract

**Objectives:** Patients with ankle fractures associated with diabetes experience more complications following standard open reduction–internal fixation (ORIF) than those without diabetes. Augmented fixation strategies, namely extended ORIF and hindfoot nails (HFNs), may offer better results and early weightbearing in this group. The aim of this study was to define the population of patients with diabetes undergoing primary fixation for ankle fractures. Secondarily, we aimed to assess the utilisation of standard and augmented strategies and the effect of these choices on surgical outcomes, including early post-operative weightbearing and surgical complications. **Methods**: A national multicentre retrospective cohort study was conducted between January and June 2019 in 56 centres (10 major trauma centres and 46 trauma units) in the United Kingdom; 1360 patients with specifically defined complex ankle fractures were enrolled. The patients’ demographics, fixation choices and surgical and functional outcomes were recorded. Statistical analysis was performed to compare high-risk patients with and without diabetes. **Results**: There were 316 patients in the diabetes cohort with a mean age of 63.9 yrs (vs. 49.3 yrs. in the non-diabetes cohort), and a greater frailty score > 4 (24% vs. 14% (non-diabetes cohort) (*p* < 0.03)); 7.5% had documented neuropathy. In the diabetes cohort, 79.7% underwent standard ORIF, 7.1% extended ORIF and 10.2% an HFN, compared to 87.7%, 3.0% and 10.3% in the non-diabetes cohort. Surgical wound complications after standard-ORIF were higher in the diabetes cohort (15.1% vs. 8.7%) (*p* < 0.02), but patients with diabetes who underwent augmented techniques showed little difference in surgical outcomes/complications compared to non-diabetes patients, even though early-weightbearing rates were greater than for standard-ORIF. **Conclusions**: Ankle fractures in diabetes occur in older, frailer patients, whilst lower-than-expected neuropathy rates suggest a need for improved assessment. Augmented surgical techniques may allow earlier weightbearing without increasing complications, in keeping with modern guidelines in ankle fracture management.

## 1. Introduction

**Figure 1:**
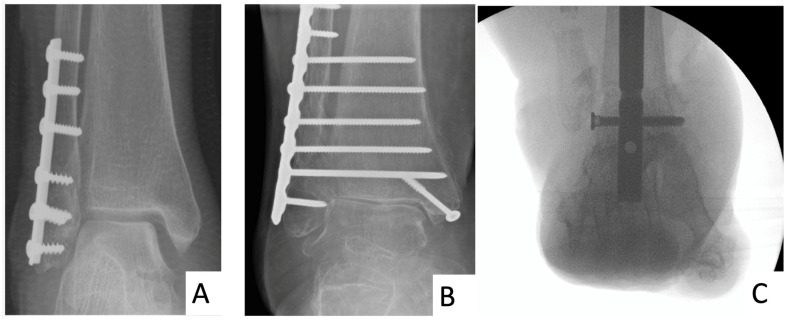
Radiographs of standard open reduction–internal fixation (**A**), extended open reduction–internal fixation (**B**) and a tibio-talar-calcaneal (hindfoot) nail (**C**).

Patients with diabetes account for 13% of those undergoing acute ankle fracture fixation. This group of patients frequently have evidence of complications of diabetes, such as peripheral neuropathy, nephropathy, peripheral arterial disease, and Charcot neuroarthropathy, which can make surgical decision-making challenging [1,2]. Non-operative management can result in catastrophic complications of immobility and pressure ulceration; thus, surgery is often indicated [3,4,5]. Unfortunately, even after ankle fracture surgery, patients with diabetes experience more complications than their non-diabetes counterparts, including impaired wound healing, malunion, non-union, soft tissue complication, and Charcot neuroarthropathy [6,7,8,9]. The infection rate in operatively treated diabetic ankle fracture can be up to 30% and is potentially higher with poorly controlled diabetes [10,11]. Furthermore, the period of post-surgical immobilisation is often extended from 6 weeks to 8–12 weeks following open reduction and internal fixation (ORIF), because of longstanding concerns regarding poor bone healing in diabetes, and the dogma “patients with diabetes take twice as long to heal” [12,13,14]. Some surgeons do not allow early weightbearing, to avoid a perceived increase in wound problems or pressure sores from the associated plaster cast [15,16]. Early weightbearing in patients with diabetes may be facilitated by two alternative (termed augmented) surgical approaches to standard ORIF. Firstly, there is “extended ORIF” which encompasses multiple plates or screws that cross from the fibula to the tibia (Figure 1) [17].

An alternative approach which permanently stabilises the hindfoot is the insertion of a hindfoot nail (HFN) or tibio-talar-calcaneal nail (TTC) [1,18]. In contrast to ORIF techniques, which utilise direct anatomic reduction and fixation with metal implants to impart absolute stability, HFN utilises indirect fracture reduction and may be used to either span the fracture or fuse the ankle joints [19].

The primary aim of this study was to compare the mode of fixation of patients undergoing primary fixation of complex ankle fractures stratified by diabetes status. The secondary aims included the assessment of the utilisation of standard and augmented strategies and clinical outcomes, including post-operative weightbearing and surgical complications.

## 2. Materials and Methods

### 2.1. Study Design

A comparative analysis of patients with and without diabetes mellitus identified as part of a national retrospective multicentre observational study was conducted using a national collaborative approach in the United Kingdom [20]. This study was reported in line with the STROBE guidelines [21].

### 2.2. Setting

This study included cases from fifty-six centres (10 major trauma centres and 46 trauma units) with representation from all 4 United Kingdom nations. Data were retrospectively collected on all patients presenting between 1st January 2019 and 30 June 2019.

### 2.3. Participants

**Figure 2:**
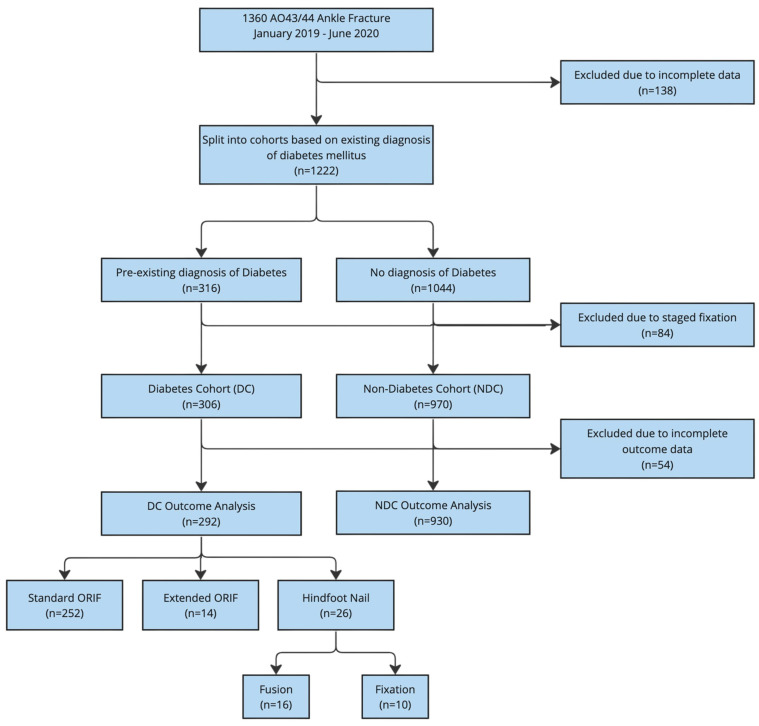
Flowchart demonstrating patient participation.

All patients aged 16 years or older who sustained complex ankle fractures involving the ankle joint (classified as an AO44/AO43 fracture where the majority of the fracture line was within one Muller square of the joint line), as seen in Figure 2, undergoing a single-stage primary definitive fixation during the study period were screened for inclusion. Patients were split into two cohorts based on whether or not they had a pre-existing diagnosis of diabetes mellitus: the diabetes cohort (DC) and the non-diabetes cohort (NDC).

Ankle fractures were defined as complex if one or more of the following were identified (see Supplementary Details S1): pre-existing or concurrent diagnoses of diabetes with or without neuropathy, rheumatoid arthritis, alcoholism, or cognitive impairment (including dementia). Fractures were also deemed complex if presenting as open fractures or associated with polytrauma (see Supplementary Details S2). Three different groups of surgical techniques were identified: standard ORIF using standard AO principles, extended ORIF and HFN. These were split into 2 surgical approaches: fixation or fusion. All patients aged under 16 years, and cases in which the fracture extended greater than one muller square from the joint, were excluded. Patients undergoing staged fixation or definitive external or frame fixation to manage soft tissue injuries were also excluded.

### 2.4. Outcome Measures

The primary outcome of this study was the mode of operative fixation stratified by diabetes status. The secondary outcomes included weightbearing status and complication rate.

### 2.5. Data Sources

Data were retrospectively obtained from each centre on patient and fracture characteristics, fixation choice and patient outcomes. Patient data included age, sex, laterality of injury, American society of Anaesthesiologists (ASA) grade and pre-operative factors (level of trauma unit receiving patient, fracture classification, open or closed fracture, polytrauma). Comorbidities were recorded and cross-referenced with the patient medical records (diabetes mellitus, peripheral neuropathy, rheumatoid arthritis, alcoholism, cognitive impairment and smoking status), abbreviated mental test score (AMTS) or prior mini mental state examination (MMSE), clinical frailty score (CFS) (see Supplementary Details S1), pre-operative mobility (e.g., unaided mobilisation, walks with one stick, two walking sticks or walking/Zimmer frame) and mental illness [22].

The operative factors were the method of definitive fixation, standard open reduction–internal fixation (ORIF), extended ORIF, augmented internal fixation, HFN and the use of external fixation/frames. The post-operative factors included immediate weightbearing status (divided into full weightbearing (FWB, i.e., all weight); partial weightbearing (PWB, i.e., where a patient is limited in the amount of weight that they can put down, e.g., toe touch); and non-weightbearing (NWB, i.e., no weight at all). Complications of surgery within 1 year were recorded, including wound breakdown, wound infection, deep vein thrombosis (DVT), pulmonary embolism (PE), failure of construct, further surgical procedures and the removal of metalwork. Patients were followed up until discharged or for a maximum of 18 months.

### 2.6. Bias

Steps were taken to reduce bias. The statistical analysis was conducted by blinded assessors and a multi-centre approach was utilised to reduce the risk of selection bias. Standardised data collection forms using clearly defined inclusion/exclusion criteria and quality assurance were utilised to minimize variations in data collection.

### 2.7. Study Size

In the design of this study, a precalculated study size was not established. The study was designed in order to assess a large number of patients in order to provide a meaningful representation of the population under study.

### 2.8. Ethical Approval and Funding

The NHS Health Research Authority decision tool was used, and this project was deemed not to be classified as clinical research requiring formal ethical approval [23]. Each centre was required to submit confirmation of local audit office approval and name a substantive consultant supervisor. There was no funding to support this study and the study lead (RA) has no conflicts of interest to declare.

### 2.9. Statistical Methods

Baseline variables are described as frequencies and percentages for categorical data and as means and SDs for continuous variables. Crude comparisons between participants with and without diabetes and outcomes of interest were assessed using an independent-samples *t*-test for continuous variables with normal distributions, whereas categorical variables were compared using a χ^2^ test. Chi-squared (χ^2^) analysis was used to assess significance between patients with diabetes and without diabetes, and subsequently, between treatment groups. Matching for baseline covariates such as age > 65 years, ASA > 3, frailty (CFS > 4) and ankle fracture type (AO43/44) allowed for a more detailed comparison of primary surgical fixation technique outcomes in patients with/without diabetes. Statistical significance was set at *p* < 0.05 for all analyses. Statistical analysis was performed using SPSS (version 26, IBM, Armonk, NY, USA).

## 3. Results

Fifty-six centres with representation from all four UK nations participated in this project. This included 10 major trauma centres (MTC) and 46 trauma units (TU) which contributed 517 and 843 cases, respectively, during the study period. Overall, this study included 1360 fractures, with complete data available for 1222 fractures (data completeness rate of 89.9%). The mean age was 53.9 years (SD +/− 19 years) with a male/female ratio of 1:1.3. In total, the median follow-up time for the reported outcomes was 7.8 months post-operatively (range of 1.2–18 months).

In total, 316 patients with ankle fractures were reported to have a pre-existing diagnosis of diabetes. Ten of these patients had either definitive external fixation or staged reconstruction to manage soft tissue injuries and were excluded from further analysis as single primary fixation was not undertaken. The baseline demographics and characteristics of each cohort can be seen in Table 1.

The primary outcome of this study was the mode of operative fixation stratified by diabetes status, as seen in Figure 2 and Table 2.

### 3.1. Standard ORIF

Wound complication and breakdown rates in the diabetes cohort were higher compared to the non-diabetes cohort (*p* < 0.003 and *p* < 0.013) (See Table 2). Wound infection rates were 31/252 (12.3%) in the diabetic cohort and 58/816 (7.6%) in the non-diabetic ankle fracture cohort (*p* = 0.022). In the standard ORIF group, metalwork removal and further surgery was more frequently performed in people with diabetes (38/252 (15.1%) vs. 87/816 (10.7%) (*p* < 0.023)). Implant failure rates were marginally greater in the cohort with diabetes (13/252 (5.3%) vs. 25/816 (3.3%) (*p* = 0.08)) although this was not statistically significant.

### 3.2. HFN

In total, 26 patients with diabetes underwent HFN, and early post-operative weightbearing was more common (76.9%; n = 20/26) (Table 3) compared to all other groups (12.2% (n = 37/303) (*p* < 0.002). Within this cohort, two groups existed due to different surgical techniques: fusion (n = 10) and fixation (n = 16). HFN fusion was observed to have a greater number of wound complications and more often required a further surgical procedure compared to HFN fixation in patients with diabetes. Overall, no significant differences in wound complication (infection or breakdown) rates or the need for further surgery and the removal of metal work were observed between the diabetes and non-diabetes cohorts undergoing HFN fixation (n = 10 vs. 62) or fusion (n = 16 vs. 23) (Table 2).

Full or partial weightbearing (FWB/PWB) with standard ORIF was observed in 14.3% (n = 36/252) of diabetes patients compared to 11.3% (n = 92/816) without diabetes (*p* = 0.192). Within the extended ORIF group, FWB or PWB was observed in 17.2% (n = 5/29) of patients without diabetes vs. 7.1% (n = 1/14) in the diabetes cohort (*p* = 0.34), but those with diabetes had higher levels of frailty (50% had a CFS greater than >4). We observed a greater percentage of PWB or FWB with/without diabetes following HFN fixation or fusion (HFN fixation: 40% (n = 4/10) with diabetes vs. 69.4% (n = 43/62) without diabetes; HFN fusion: 50% (n = 8/16) with diabetes vs. 73.9% (n = 17/23) without diabetes. Thus, the highest rate of PWB or FWB in patients with diabetes was seen following HFN, irrespective of technique (70.7% (n = 20/26)) compared to standard ORIF (14% (36/252) (*p* < 0.002)) or extended ORIF (7.1% (1/14) (*p* < 0.001)).

Adjusting for age (>65) and ASA (1/2 vs. 3/4 patients), we compared techniques and functional and surgical outcomes between those patients considered to be high risk (age > 65, and ASA 3/4) with and without diabetes. In total n = 116/252 patients with an ASA of 3/4 and an age >65 and diabetes had standard ORIF fixation, and they were found to be more likely to be non-weightbearing in the immediate post-operative period (*p* < 0.03) and had a higher prevalence of wound breakdown (*p* < 0.016) compared to those without diabetes (n = 146/812). These differences in complication rates were not observed when comparing those with diabetes and without diabetes who underwent extended ORIF and/or either HFN technique, whilst more patients in these groups were partial weightbearing or full weightbearing in the immediate post-operative period (*p* < 0.04).

Independent of surgery type and diabetes status, patients were more likely to be weightbearing if treated in a Major Trauma Centre (MTC) as opposed to a Trauma unit (17.6% vs. 3.8%) (Table 3). HFN was equally likely to be performed at an MTC or a trauma unit, with a greater tendency to allow either partial weightbearing or full weightbearing than those fixed with extended fixation techniques (Table 3). There were no observable differences in complications rates, treatment times and outcomes between the two types of institution.

## 4. Discussion

In this study of complex ankle fractures treated with primary internal fixation, we observed that the diabetes cohort was frailer and older with poorer pre-operative mobility compared with non-diabetes patients. Those with diabetes were also more likely to have sustained an ankle fracture without fracture extension into the tibial shaft compared to those without diabetes, in keeping with low-velocity trauma. The incidence of peripheral neuropathy was higher in diabetic patients. Contrary to current guidelines, most patients were non-weightbearing post-surgery.

### 4.1. The Significance of Diabetes in the Surgical Management of Ankle Fractures

After surgery, the ORIF group exhibited higher rates of surgical complications when compared to those without diabetes, whereas the augmented-fixation groups did not experience such an increase. Furthermore, after standard ORIF, wound infection rates were higher in the cohort with diabetes (12.3% vs. 7.6%). Wukich et al. assessed 1000 patients undergoing elective surgery and found a similar surgical site infection rate of 13.2% in diabetes, compared to 2.8% in non-diabetes patients [24]. SooHoo et al. (2009) reported that the odds ratio of major amputations in diabetes patients following ankle ORIF was extremely high (3.86% compared to 0.16% in non-diabetes patients—resulting in an OR of 27.6) [25]. However, no amputations were recorded in the present study—this could be due to the median follow-up of 7 months.

### 4.2. The Need for a Multi-Disciplinary Approach

This study has demonstrated that the diabetic population undergoing primary fixation is older and frailer and thus would benefit from multidisciplinary care, ranging from inpatient diabetic foot care to treatment by surgeons. As many still need long-term orthotics or bracing or appropriate on-going glycaemic control and prevention of diabetes foot syndrome, multidisciplinary care has become more important [26]. Early MDT input is necessary and acute vascular support may be needed if peripheral macro-angiopathy is suspected [6,27,28,29].

### 4.3. Quantifying the Extent of Diabetes Complications

Multidisciplinary involvement would also facilitate the assessment of diabetic complications in patients with diabetes presenting with an ankle fracture. It is important to know the extent of diabetic complications, especially peripheral neuropathy, as it is associated with surgical complications, longer in-hospital stays and increased costs of foot and ankle surgery compared to those without diabetes [6,7,8,9].

Low rates of peripheral neuropathy in diabetes were reported in the present study. This was surprising, as peripheral neuropathy and its prevalence has been reported to be as high as 51% in patients with diabetes [30]. It is therefore likely that this finding was under-reported. A recent audit indicated significant variation in practice in the clinical documentation of neurovascular status, despite current best practice guidelines in ankle fracture management advising examination for peripheral neuropathy [27,31,32]. No standardisation of processes or guidance in measuring it in acute fractures currently exists. The detection of diabetic peripheral neuropathy is challenging when a fracture is rapidly immobilised in a plaster to prevent further injury [33,34].

The detection of peripheral neuropathy may help to determine the mode of surgical fixation by prompting the use of augmented fixation techniques which can promote early mobilisation and avoid the higher complication rates seen with standard ORIF [1]. Meanwhile, as the severity of neuropathy is directly related to poor glycaemic control, HbA1C maybe a substitute marker [35,36]. Level I evidence has shown surgical site infections to be independently associated with both peripheral neuropathy and HbA1C > 8%, whilst an HbA1C value greater than 6.5% in diabetic patients sustaining ankle fractures has been correlated with poorer radiological and clinical outcomes [37,38].

### 4.4. Challenging Surgical Dogma: The Potential of HFN and Extended ORIF in Limiting Post-Operative Immobilisation/Non-Weightbearing in Diabetes

Diabetes is associated with slower fracture healing, which traditionally leads to prolonged immobilization with restricted weightbearing [4,7,10]. No weightbearing increases the risk of pressure sores, pneumonia and venous-thromboembolic events. The short-term surgical outcomes of extended ORIF fixation strategies and HFN were similar in both diabetic and non-diabetic patients. However, the need for further surgery was highest in the HFN-fusion group. Although the utilisation of augmented techniques may not always allow earlier weightbearing, it may confer the ability to stand and transfer in this frail and elderly cohort with diabetes, akin to the benefits of early mobilisation demonstrated in geriatric hip fracture treatment [35,39,40,41,42]. The comparison of surgical techniques here shows that the utilisation of extended-ORIF/HFN techniques provided no difference in complication rates between the high (ASA3/4)- and low-risk groups (ASA1/2) with or without diabetes. This suggests that these techniques are safe in all populations and reduce the risk of complications when compared to standard ORIF to allow early mobilisation [41]. There is a need for further studies with detailed data to perform logistic regression to find the strength of association of the risk factors with outcomes in each surgery.

### 4.5. Limitations

Notable limitations of a retrospective study include selection and reporting bias. Surgical decision making, particularly with regard to the choice of HFN vs. internal fixation, may have been determined by factors including clinical knowledge and experience and soft tissue status, which were not captured. Complications are likely to be under-reported due to variations in clinical documentation and follow-up patterns. Larger numbers are required to exclude the effects of confounding factors and provide statistical significance in assessing individual inclusion criteria and the effects of prolonged protected weightbearing of the different surgical techniques. The lack of functional outcome measures and biochemical markers (HbA1C) and limited follow-up length limits the generalisability of our results and does not provide a long-term assessment of the treatment arms. Further studies are required to develop appropriate algorithms for patient selection and understand surgical choices, especially if joints are being immobilised. The key aim of this study was to understand the current state of practice in the UK with regard to these complex fractures in order to guide the design and development of future studies. It is important for readers to note that this study did not control or report other variables that can affect clinical outcomes, such as the use of corticosteroids, antibiotic prophylaxis or the method of anaesthesia.

## 5. Conclusions

Ankle fractures in diabetes occur more often in older and frailer patients with higher surgical complication rates with standard fixation techniques compared to similar patients without diabetes. A multidisciplinary approach similar to the treatment of hip fractures incorporating orthogeriatricians and diabetic foot teams should be adopted. Careful assessment of neuropathy and other known risk factors guide surgical decision making regarding augmented fixation techniques, which may facilitate early weightbearing.

## Figures and Tables

**Table 1:** An overall summary table of the characteristics of patients with complex ankle fractures comparing characteristics in a group of patients with diabetes (n = 306) and a control group of patients without diabetes (n = 970).

		Diabetes Cohort (N = 306)	Non-Diabetes Cohort (N = 970)	*p* Value
Age (Mean ± SD)		63.89 ± 15.27	49.37 ± 19.61	<0.001
Gender	Male	111 (36.3%)	499 (51.4%)	<0.001
	Female	195 (63.7%)	471 (48.6%)	
Anatomic Descriptor	AO43	25 (8.2%)	336 (34.6%)	<0.001
	AO44	281 (91.8%)	634 (65.4%)	
Clinical Frailty (Score > 4)		100 (32.7%)	146 (15%)	<0.001
ASA Grade	Median [IR]	3 [2–3]	2 [1–2]	<0.001
	I	7 (2.3%)	334 (34.4%)	<0.001
	II	132 (43.1%)	397 (40.9%)	0.363
	III	157 (51.3%)	192 (19.7%)	<0.001
	IV	10 (3.3%)	39 (4.2%)	0.006
	V	0	10 (1.0%)	
Pre-Operative Status	Rheumatoid Arthritis	3 (1%)	16 (1.6%)	0.338
	Alcoholism	23 (7.5%)	169 (17.4%)	<0.001
	Peripheral neuropathy	23 (7.5%)	22 (2.3%)	<0.001
	Mental health	12 (3.9%)	103 (10.6%)	0.01
	Dementia	9 (2.9%)	34 (3.6%)	0.574
	Smoker	43 (14.1%)	245 (25.2%)	<0.001
Pre-Operative Mobility	Unaided mobilisation	232 (75.8%)	835 (86.1%)	<0.001
	Walks with one stick	47 (15.3%)	45 (4.6%)	<0.001
	Two walking sticks or walking/Zimmer frame	27 (8.8%)	54 (5.6%)	0.054
	Non-weightbearing/wheelchair	11 (3.6%)	8 (0.8%)	0.001

**Table 2:** Comparison of fixation technique-specific outcomes and association of diabetes and non-diabetes groups.

Fixation Type and Diabetes Group		Standard ORIF		Extended ORIF		HFN (Fixation)		HFN (Fusion)	
		DC (n = 252)	NDC (n = 816)	DC (n = 14)	NDC (n = 29)	DC (n = 10)	NDC (n = 62)	DC (n = 16)	NDC (n = 23)
Post-operative weightbearing status	Non-weightbearing	216 (85.7%)	724 (88.7%)	13 (92.9%)	24 (82.8%)	14 (53.4%) **	18 (29%)	8 (50%) *	6 (26.1%)
	Partially weightbearing	20 (7.9%)	61 (7.5%)	1 (7.1%)	4 (13.8%)	3 (11.5%)	20 (32.3%)	2 (12.5%)	10 (43.5%) **
	Fully weightbearing	16 (6.3%)	31 (7.5%)	0	1 (3.4%)	9 (34.6%)	23 (37.1%)	6 (37.5%)	7 (30.4%)
Post-operative Complications	Wound complication	38 (15.1%)	71 (8.7%) *	2 (14.3%)	3 (10.3%)	1 (10%)	2 (3.3%)	5 (33.3%)	3 (13%)
	Wound breakdown	24 (9.1%) *	40 (5.3%) *	0	3 (10.3%)	0	0	5 (31.3%)	3 (13%) **
	Wound infection	31 (12.3%) *	58 (7.6%) *	2 (14.3%)	3 (10.3%)	1 (10%)	2 (3.3%)	3 (18.8%)	4 (17.4%)
	DVT	0	4 (0.6%)	0	0	0	0	1 (6.3%)	0
	PE	2 (0.8%)	4 (0.5%)	0	0	0	0	1 (6.3%)	1 (4.3%)
	Further procedure	24 (9.5%)	66 (8.7%)	3 (21.4%)	4 (13.8%)	0	3 (4.9%)	5 (31.3%)	3 (13%)
	Failure of construct	13 (5.3%)	25 (3.3%) **	1 (7.1%)	2 (6.9%)	0	0	1 (6.3%)	2 (9.1%)
	Removal of metalwork	25 (10.3%)	62 (8.5%)	3 (21.4%)	4 (13.8%)	0	2 (3.5%)	3 (18.8%)	4 (18.2%)

DC: diabetes cohort, NDC: non-diabetes cohort. * Observed statistical difference (*p* < 0.05) between diabetes and non-diabetes groups for said technique; ** observed difference indicative of a trend between diabetes and non-diabetes groups for said technique (*p* < 0.06–0.08). Analysis was undertaken using Chi-square test for cells with values > 5. If value of a cell was <5, Fisher’s Exact test was used.

**Table 3:** Clinical outcomes following primary fixation in patients with a complex ankle fracture.

		Standard ORIF (n = 252)	Extended ORIF (n = 14)	HFN Fixation (n = 10)	HFN Fusion (n = 16)
Post-operative Weightbearing Status	Non-weightbearing	216 (85.7%)	13 (92.9%)	6 (60%)	8 (50%)
	Partially weightbearing	20 (7.9%)	1 (7.1%)	1 (10%)	2 (12.5%)
	Fully weightbearing	16 (6.3%)	0	3 (30%)	6 (37.5%)
Complication	Wound complication	38 (15.1%)	2 (14.3%)	1 (10%)	6 (37.5%)
	DVT	0	0	0	1 (6.3%)
	PE	2 (0.8%)	0	0	1 (6.3%)
	Further procedure	24 (9.5%)	3 (21.4%)	0	5 (31.3%)
	Failure of construct	13 (5.3%)	1 (7.1%)	0	1 (6.3%)
	Removal of metalwork	25 (10.3%)	3 (21.4%)	0	3 (18.8%)

## Data Availability

Data are contained within the article.

## Supplementary Materials

The following supporting information can be downloaded at: https://www.mdpi.com/article/10.3390/jcm13133949/s1, Supplementary Details S1: Details regarding definition of ‘Complex Ankle Fracture’; Supplementary Details S2: Patient data Collection Tool and associated guidance.
