# Supplementary material for: Surgical Management of Complex Ankle Fractures in Patients with Diabetes: A National Retrospective Multicentre Study
Source: J Clin Med. 2024 Jul 5;13(13):3949. doi: 10.3390/jcm13133949 (PMC11242888; doi:10.3390/jcm13133949)
Supplement: Supplementary file 1 [file jcm-13-03949-s001.zip › jcm-2894384-supplementary.pdf]

Supplementary Details S1

'Complex Ankle fractures' were defined following discussion and literature review within the scientific steering group and representatives from the OTS (orthopaedic Trauma Society) and BOTA (British Orthopaedic Trainee Association). In addition to age, various patient factors also impact upon the successful treatment of these injuries, including fracture configuration, medical comorbidities, bone quality, soft tissue condition, functional and cognitive baseline. Whilst previously identified risk factors for poor outcomes in patients with ankle fractures, regardless of treatment type include, high body mass index, diabetes mellitus, peripheral neuropathy, mental illness and walking aids.

Supplementary Details S2– **Final data collection tool [online publication only]**

<u>Patient data collection tool guidance</u>

<u>Section SA – demographics:</u>
Hospital: Hospital Abbreviation [see below]
Study ID: [Hospital name] followed by 1/2/3/4 sequence
Receiving unit level – MTC/Designated Trauma unit/ DGH
Transferred to major trauma centre – yes/no
Age: to nearest whole year
Sex: Male / Female / Prefers to self-describe
Injury side: Right/Left (bilateral injuries as 2 separate rows in spreadsheet with different study ID's)
Time & Date of Diagnosis from the initial imaging: Recorded as dd/mm/yy, hh:mm (24hr clock)

<u>Section SB - Fracture Classification:</u>

DO NOT INCLUDE NON-COMPLEX FRACTURES – for this study only include AO43 or 44 fractures with <u>one or more</u> of the following patient or fracture characteristics:

- Open fracture
- Diagnosis of diabetes mellitus – type I or type II
- Diagnosis of peripheral neuropathy
- Diagnosis of alcoholism
- Polytrauma patient with high-dependency or intensive care admission
- Reduced cognition or mental function that would result in reduced ability to follow non or partial weight bearing instructions

AO 43: _____________

Main fracture line must be within one Muller square of tibial plafond to be included (excluded tibial shaft)

Or, AO 44: _________

Other hindfoot fracture of ipsilateral side: calcaneus or talus.
Open/Closed Injury: yes/no/unknown
Gustillo Anderson classification
If pilon / plafond AO43, does the majority of fracture line occur within on muller square of the tibial plafond? Yes/No – if not, we do not want these fractures included, (i.e., diaphyseal with metaphyseal extension)

<u>Section S3 – Management:</u>
Polytrauma: yes/no

- **Polytrauma definition:** complex ankle fracture PLUS one or more of the following:
  - Bilateral lower limb fractures
  - Upper limb fracture (excluding the hand)
  - Pelvic fracture
  - Chest injury (rib fractures, scapula fractures, or any intra-thoracic injury to the chest that requires intervention)
  - Intra-abdominal injury
  - Head injury (intra-cranial event, or fracture to the skull or facial bones)
  - Spinal injury (an injury to the spinal cord OR spinal fracture/spinal ligamentous injury that requires operative management or restriction to weight bearing / mobility of the spine)

Guidance: Most of this information is found in electronic patient records and PACS systems. You may also find the following useful sources of information may be sought from theatre lists, theatre logbooks, trauma admission database and any additional imaging conducted on the patient

Intensive care / High dependency unit admission pre-operatively:
Yes/ No

Initial procedures-
Open fractures – date and time of 1st debridement
Time to initial debridement – nearest whole hour
Number of procedures before definitive surgery

Time & Date of Definitive Surgery for acute fracture:
Recorded as dd/mm/yy, hh:mm (24hr clock).

ASA grade

Definitive surgical procedure:
I) ORIF: plate and screw fixation or fibula nail
II) Nail: Hind foot nail/ Femoral/ Tibial Nail
III) External fixator: ex-fix, ilizarov, or taylor spatial frame

Specify Type and length of nail and /or implants used

Method of joint preparation (nail only):

- All joints prepared for fusion
- Only ankle joint prepared
- No joints prepared

<u>Section S4 - patient factors:</u>

Patients Co-morbidities: Yes/No only
Diabetes mellitus
Rheumatoid arthritis
Alcoholism
Peripheral neuropathy of other cause
Smoker
Mental health diagnosis (except dementia)
Dementia

AMTS:
___ / 10 or, NOT recorded

Clinical Frailty Scale Score (see below)**:**
___ / 10

In most cases, this can be calculated from review of medical notes and social history.

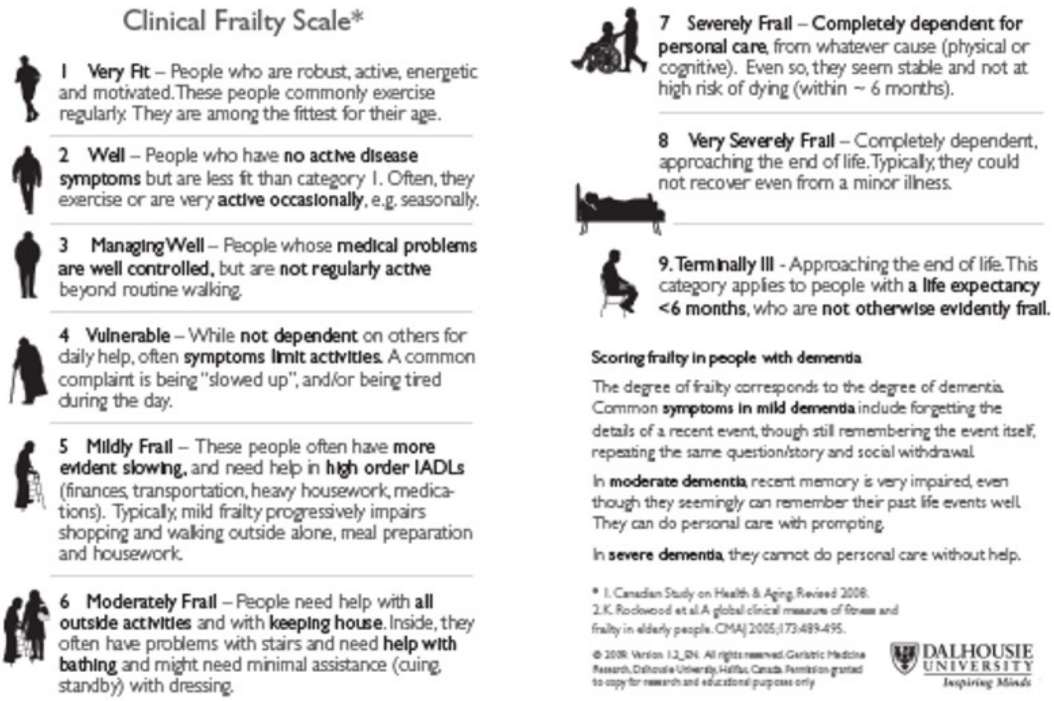

Clinical Frailty Scale*

1 **Very Fit** – People who are robust, active, energetic and motivated. These people commonly exercise regularly. They are among the fittest for their age.

2 **Well** – People who have **no active disease symptoms** but are less fit than category 1. Often, they exercise or are very **active occasionally**, e.g. seasonally.

3 **Managing Well** – People whose **medical problems are well controlled**, but are **not regularly active** beyond routine walking.

4 **Vulnerable** – While **not dependent** on others for daily help, often **symptoms limit activities**. A common complaint is being "slowed up", and/or being tired during the day.

5 **Mildly Frail** – These people often have **more evident slowing**, and need help in **high order IADLs** (finances, transportation, heavy housework, medications). Typically, mild frailty progressively impairs shopping and walking outside alone, meal preparation and housework.

6 **Moderately Frail** – People need help with **all outside activities** and with **keeping house**. Inside, they often have problems with stairs and need **help with bathing** and might need minimal assistance (cuing, standby) with dressing.

7 **Severely Frail** – **Completely dependent for personal care**, from whatever cause (physical or cognitive). Even so, they seem stable and not at high risk of dying (within ~ 6 months).

8 **Very Severely Frail** – Completely dependent, approaching the end of life. Typically, they could not recover even from a minor illness.

9. **Terminally Ill** - Approaching the end of life. This category applies to people with **a life expectancy <6 months**, who are **not otherwise evidently frail.**

Scoring frailty in people with dementia

The degree of frailty corresponds to the degree of dementia. Common **symptoms in mild dementia** include forgetting the details of a recent event, though still remembering the event itself, repeating the same question/story and social withdrawal.

In **moderate dementia**, recent memory is very impaired, even though they seemingly can remember their past life events well. They can do personal care with prompting.

In **severe dementia**, they cannot do personal care without help.

* 1. Canadian Study on Health & Aging, Revised 2008.
2. K. Rockwood et al. A global clinical measure of fitness and frailty in elderly people. CMAJ 2005;173:489-495.

© 2008. Version 1.2_EN. All rights reserved. Geriatric Medicine Research, Dalhousie University, Halifax, Canada. Permission granted to copy for research and educational purposes only

DALHOUSIE UNIVERSITY Inspiring Minds

Reproduced with permission: REF: Rockwood K, Song X, MacKnight C, Bergman H, Hogan DB, McDowell I, Mitnitski A. A global clinical measure of fitness and frailty in elderly people. CMAJ. 2005 Aug 30;173(5):489-95

Patients pre-injury weight bearing status:
Unaided mobilisation
Walks with one stick
Two walking sticks or walking/zimmer frame
Wheelchair bound

Pre-injury residence:
Own Home
Warden assisted complex
Nursing or care home resident

Patients immediate post-operative weight bearing status:
Non-weight bearing
Partial weight bearing (including toe-touch)
Fully weight bearing

<u>Section S5 – outcomes:</u>

Discharge destination:
Pre-injury residence / rehab unit / local hospital

Length of stay in hospital:
___ Days (whole days)

Complications within 12 months of surgery:
Wound breakdown: yes / no
Wound infection: yes / no
Deep vein thrombosis: yes / no
Pulmonary embolus: yes / no
Further surgical procedure: yes / no
Failure of construct: yes / no
Removal of metalwork: yes / no

If managed with ORIF – was hindfoot nail used as a salvage procedure?
Yes/No

Outcome at 6 and at 12 months post-injury:

- Fracture or prepared joint united
- Fracture site or prepared joint non-union / malunion
- Not available / recorded

Date of last (most recent) clinic review:
Date

Please include any free text or comments in the final column you wish to add.
